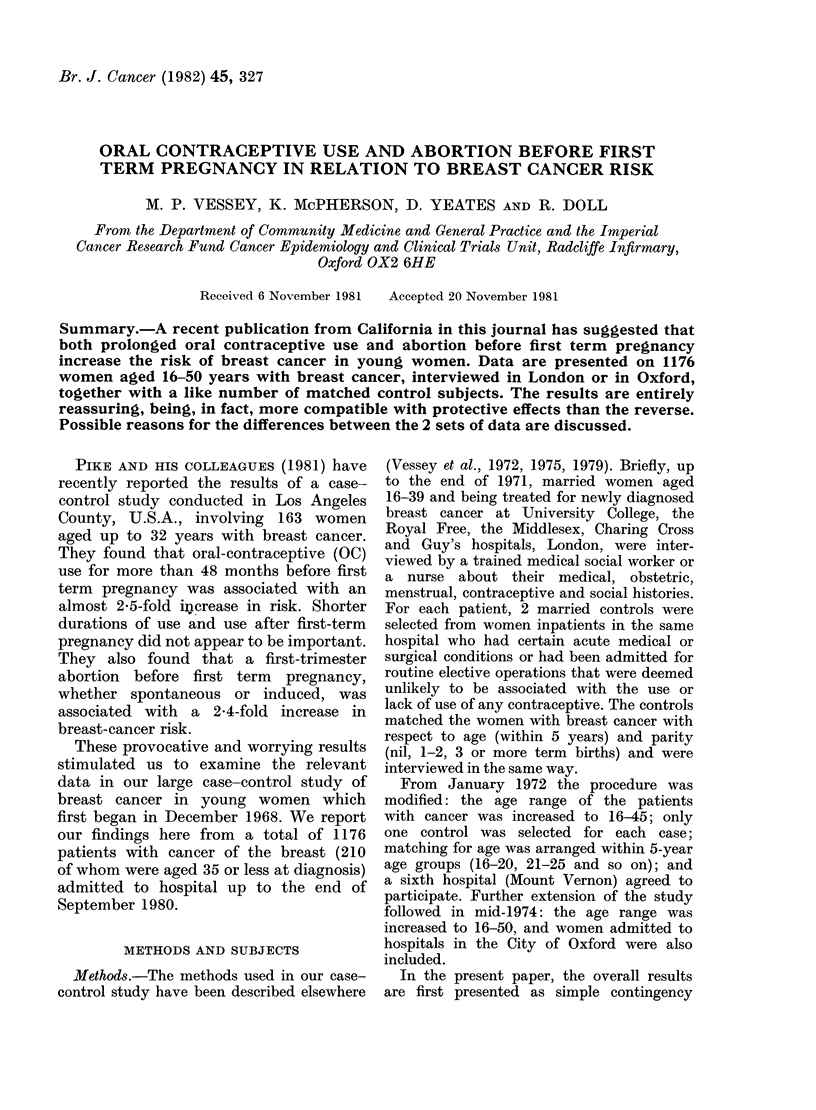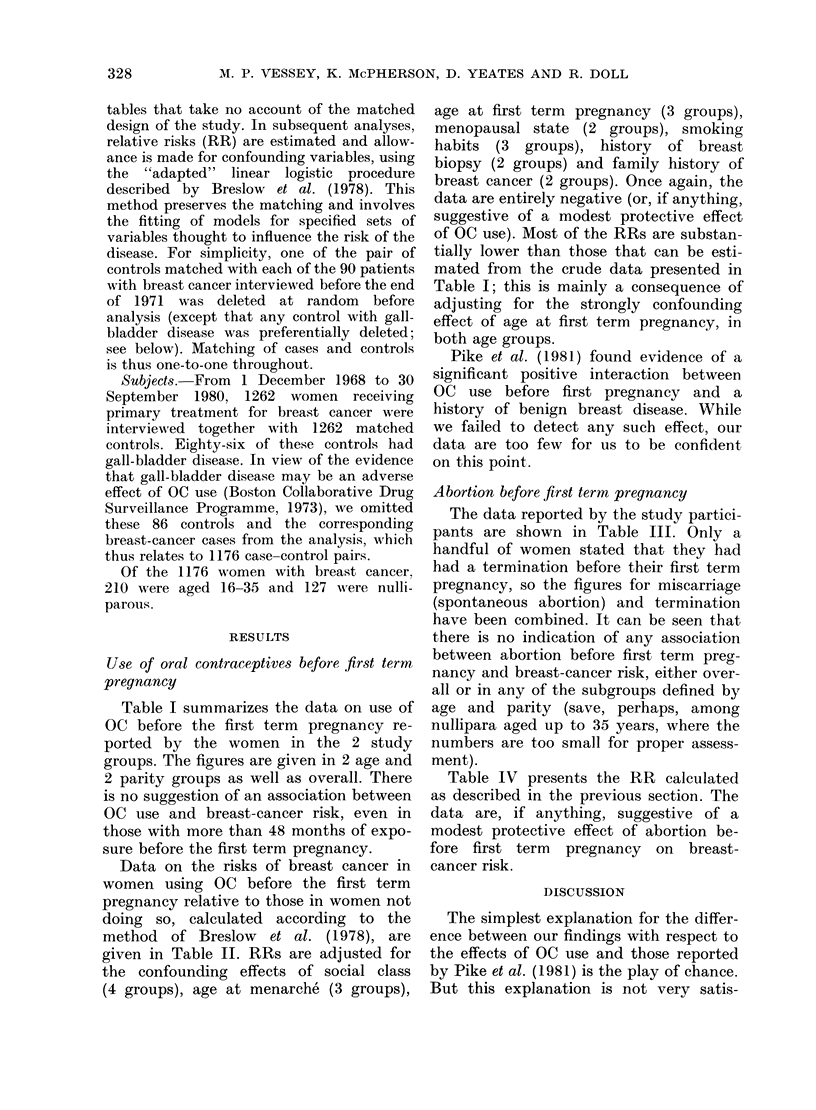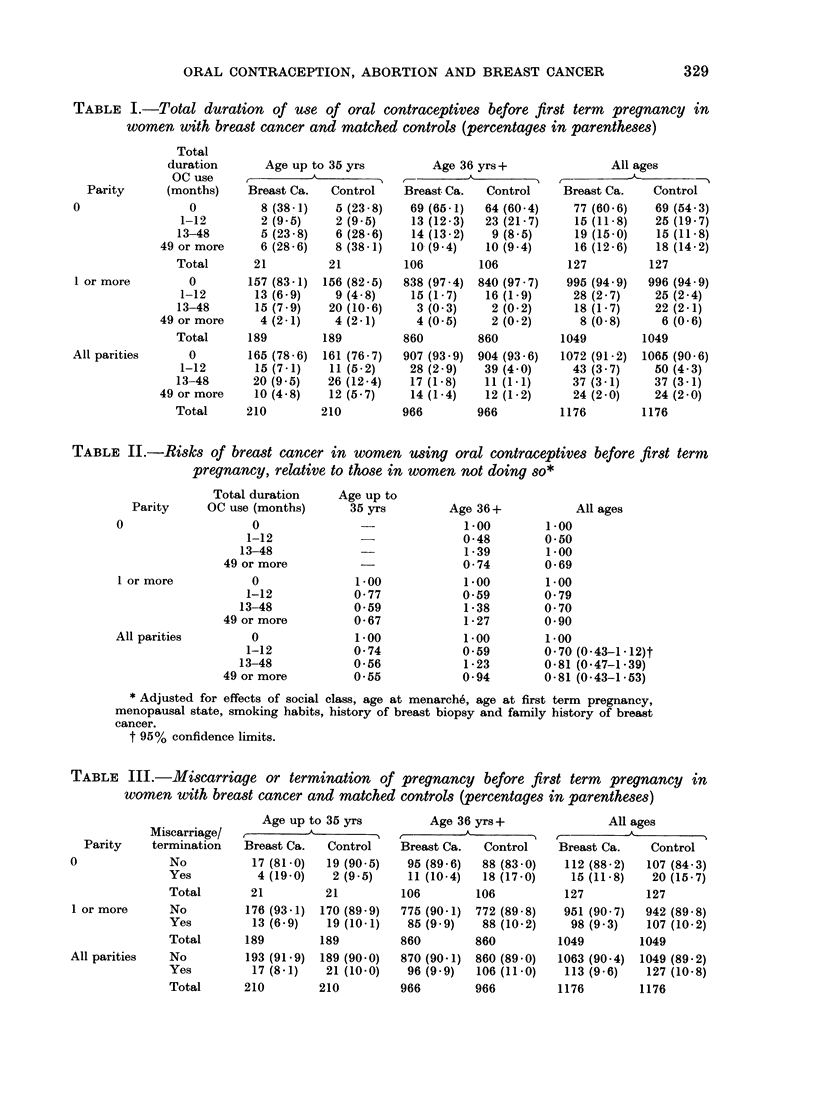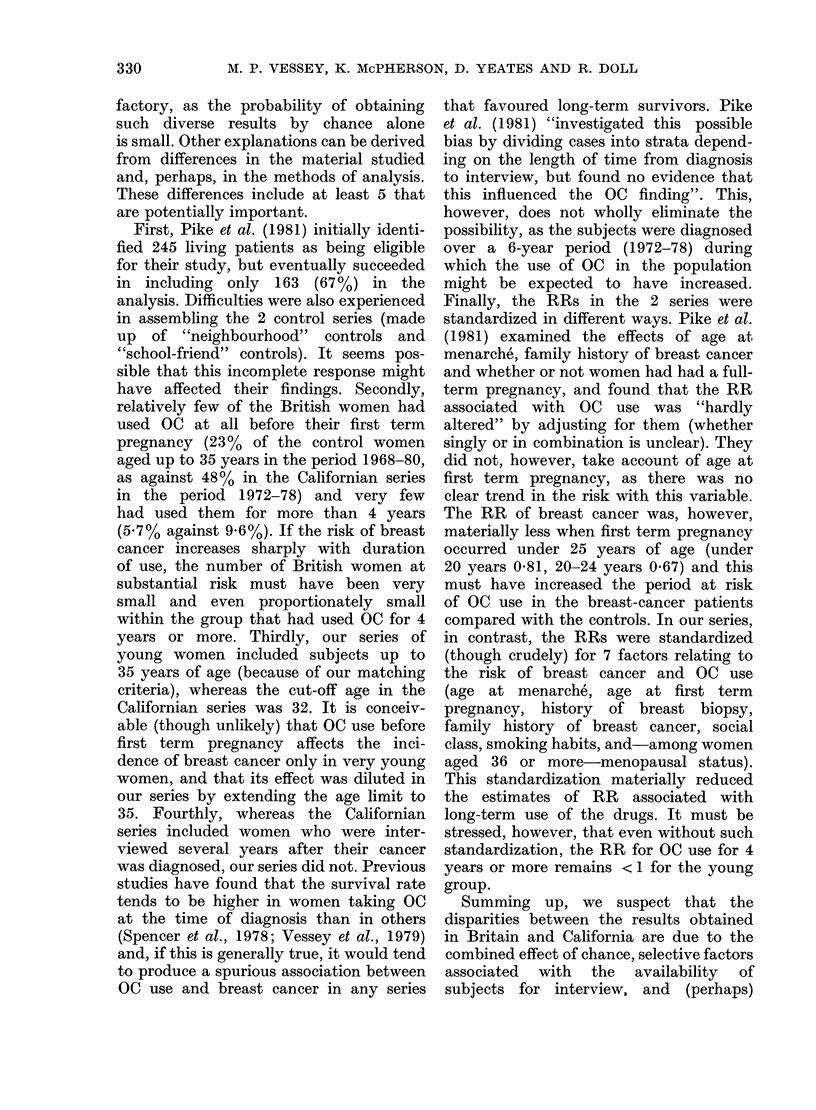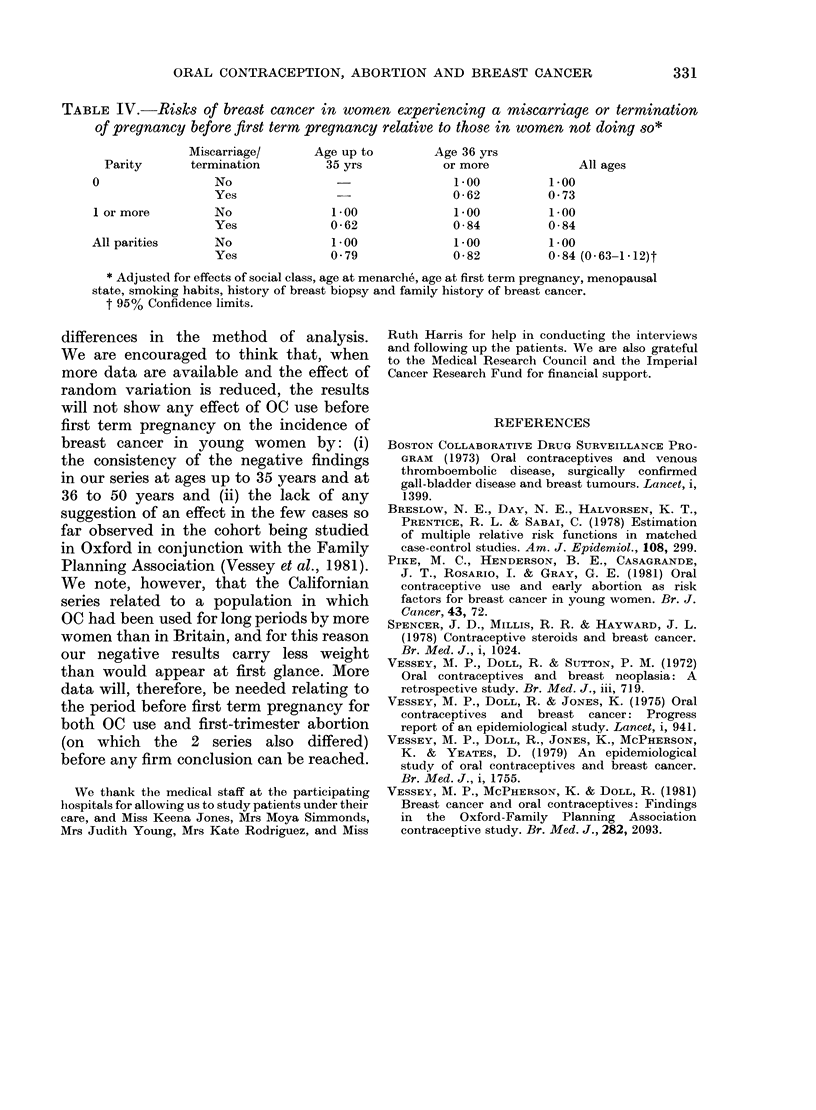# Oral contraceptive use and abortion before first term pregnancy in relation to breast cancer risk.

**DOI:** 10.1038/bjc.1982.58

**Published:** 1982-03

**Authors:** M. P. Vessey, K. McPherson, D. Yeates, R. Doll

## Abstract

A recent publication from California in this journal has suggested that both prolonged oral contraceptive use and abortion before first term pregnancy increases the risk of breast cancer in young women. Data are presented on 1176 women aged 16-50 years with breast cancer, interviewed in London or in Oxford, together with a like number of matches control subjects. The results are entirely reassuring, being, in fact, more compatible with protective effects than the reverse. Possible reasons for the differences between the 2 sets of data are discussed.


*Br. J. Cancer* (1982) **45**, 327

### ORAL CONTRACEPTIVE USE AND ABORTION BEFORE FIRST TERM PREGNANCY IN RELATION TO BREAST CANCER RISK

M. P. VESSEY, K. McPHERSON, D. YEATES AND R. DOLL

*From the Department of Community Medicine and General Practice and the Imperial Cancer Research Fund Cancer Epidemiology and Clinical Trials Unit, Radcliffe Infirmary, Oxford OX2 6HE*

Received 6 November 1981 Accepted 20 November 1981

**Summary.—A recent publication from California in this journal has suggested that both prolonged oral contraceptive use and abortion before first term pregnancy increase the risk of breast cancer in young women. Data are presented on 1176 women aged 16–50 years with breast cancer, interviewed in London or in Oxford, together with a like number of matched control subjects. The results are entirely reassuring, being, in fact, more compatible with protective effects than the reverse. Possible reasons for the differences between the 2 sets of data are discussed.**

PIKE AND HIS COLLEAGUES (1981) have recently reported the results of a case–control study conducted in Los Angeles County, U.S.A., involving 163 women aged up to 32 years with breast cancer. They found that oral-contraceptive (OC) use for more than 48 months before first term pregnancy was associated with an almost 2·5-fold increase in risk. Shorter durations of use and use after first-term pregnancy did not appear to be important. They also found that a first-trimester abortion before first term pregnancy, whether spontaneous or induced, was associated with a 2·4-fold increase in breast-cancer risk.

These provocative and worrying results stimulated us to examine the relevant data in our large case–control study of breast cancer in young women which first began in December 1968. We report our findings here from a total of 1176 patients with cancer of the breast (210 of whom were aged 35 or less at diagnosis) admitted to hospital up to the end of September 1980.

### METHODS AND SUBJECTS

*Methods.*—The methods used in our case–control study have been described elsewhere (Vessey *et al.*, 1972, 1975, 1979). Briefly, up to the end of 1971, married women aged 16–39 and being treated for newly diagnosed breast cancer at University College, the Royal Free, the Middlesex, Charing Cross and Guy's hospitals, London, were interviewed by a trained medical social worker or a nurse about their medical, obstetric, menstrual, contraceptive and social histories. For each patient, 2 married controls were selected from women inpatients in the same hospital who had certain acute medical or surgical conditions or had been admitted for routine elective operations that were deemed unlikely to be associated with the use or lack of use of any contraceptive. The controls matched the women with breast cancer with respect to age (within 5 years) and parity (nil, 1–2, 3 or more term births) and were interviewed in the same way.

From January 1972 the procedure was modified: the age range of the patients with cancer was increased to 16–45; only one control was selected for each case; matching for age was arranged within 5-year age groups (16–20, 21–25 and so on); and a sixth hospital (Mount Vernon) agreed to participate. Further extension of the study followed in mid-1974: the age range was increased to 16–50, and women admitted to hospitals in the City of Oxford were also included.

In the present paper, the overall results are first presented as simple contingency tables that take no account of the matched design of the study. In subsequent analyses, relative risks (RR) are estimated and allowance is made for confounding variables, using the "adapted" linear logistic procedure described by Breslow *et al.* (1978). This method preserves the matching and involves the fitting of models for specified sets of variables thought to influence the risk of the disease. For simplicity, one of the pair of controls matched with each of the 90 patients with breast cancer interviewed before the end of 1971 was deleted at random before analysis (except that any control with gall-bladder disease was preferentially deleted; see below). Matching of cases and controls is thus one-to-one throughout.

*Subjects.*—From 1 December 1968 to 30 September 1980, 1262 women receiving primary treatment for breast cancer were interviewed together with 1262 matched controls. Eighty-six of these controls had gall-bladder disease. In view of the evidence that gall-bladder disease may be an adverse effect of OC use (Boston Collaborative Drug Surveillance Programme, 1973), we omitted these 86 controls and the corresponding breast-cancer cases from the analysis, which thus relates to 1176 case–control pairs.

Of the 1176 women with breast cancer, 210 were aged 16–35 and 127 were nulliparous.

### RESULTS

### *Use of oral contraceptives before first term pregnancy*

Table I summarizes the data on use of OC before the first term pregnancy reported by the women in the 2 study groups. The figures are given in 2 age and 2 parity groups as well as overall. There is no suggestion of an association between OC use and breast-cancer risk, even in those with more than 48 months of exposure before the first term pregnancy.

Data on the risks of breast cancer in women using OC before the first term pregnancy relative to those in women not doing so, calculated according to the method of Breslow *et al.* (1978), are given in Table II. RRs are adjusted for the confounding effects of social class (4 groups), age at menarché (3 groups), age at first term pregnancy (3 groups), menopausal state (2 groups), smoking habits (3 groups), history of breast biopsy (2 groups) and family history of breast cancer (2 groups). Once again, the data are entirely negative (or, if anything, suggestive of a modest protective effect of OC use). Most of the RRs are substantially lower than those that can be estimated from the crude data presented in Table I; this is mainly a consequence of adjusting for the strongly confounding effect of age at first term pregnancy, in both age groups.

Pike *et al.* (1981) found evidence of a significant positive interaction between OC use before first pregnancy and a history of benign breast disease. While we failed to detect any such effect, our data are too few for us to be confident on this point.

### *Abortion before first term pregnancy*

The data reported by the study participants are shown in Table III. Only a handful of women stated that they had had a termination before their first term pregnancy, so the figures for miscarriage (spontaneous abortion) and termination have been combined. It can be seen that there is no indication of any association between abortion before first term pregnancy and breast-cancer risk, either overall or in any of the subgroups defined by age and parity (save, perhaps, among nullipara aged up to 35 years, where the numbers are too small for proper assessment).

Table IV presents the RR calculated as described in the previous section. The data are, if anything, suggestive of a modest protective effect of abortion before first term pregnancy on breast-cancer risk.

### DISCUSSION

The simplest explanation for the difference between our findings with respect to the effects of OC use and those reported by Pike *et al.* (1981) is the play of chance. But this explanation is not very satis-

TABLE I.—*Total duration of use of oral contraceptives before first term pregnancy in women with breast cancer and matched controls (percentages in parentheses)*

| Parity | Total duration OC use (months) | Age up to 35 yrs: Breast Ca. | Age up to 35 yrs: Control | Age 36 yrs+: Breast Ca. | Age 36 yrs+: Control | All ages: Breast Ca. | All ages: Control |
|---|---|---|---|---|---|---|---|
| 0 | 0 | 8 (38·1) | 5 (23·8) | 69 (65·1) | 64 (60·4) | 77 (60·6) | 69 (54·3) |
| | 1–12 | 2 (9·5) | 2 (9·5) | 13 (12·3) | 23 (21·7) | 15 (11·8) | 25 (19·7) |
| | 13–48 | 5 (23·8) | 6 (28·6) | 14 (13·2) | 9 (8·5) | 19 (15·0) | 15 (11·8) |
| | 49 or more | 6 (28·6) | 8 (38·1) | 10 (9·4) | 10 (9·4) | 16 (12·6) | 18 (14·2) |
| | Total | 21 | 21 | 106 | 106 | 127 | 127 |
| 1 or more | 0 | 157 (83·1) | 156 (82·5) | 838 (97·4) | 840 (97·7) | 995 (94·9) | 996 (94·9) |
| | 1–12 | 13 (6·9) | 9 (4·8) | 15 (1·7) | 16 (1·9) | 28 (2·7) | 25 (2·4) |
| | 13–48 | 15 (7·9) | 20 (10·6) | 3 (0·3) | 2 (0·2) | 18 (1·7) | 22 (2·1) |
| | 49 or more | 4 (2·1) | 4 (2·1) | 4 (0·5) | 2 (0·2) | 8 (0·8) | 6 (0·6) |
| | Total | 189 | 189 | 860 | 860 | 1049 | 1049 |
| All parities | 0 | 165 (78·6) | 161 (76·7) | 907 (93·9) | 904 (93·6) | 1072 (91·2) | 1065 (90·6) |
| | 1–12 | 15 (7·1) | 11 (5·2) | 28 (2·9) | 39 (4·0) | 43 (3·7) | 50 (4·3) |
| | 13–48 | 20 (9·5) | 26 (12·4) | 17 (1·8) | 11 (1·1) | 37 (3·1) | 37 (3·1) |
| | 49 or more | 10 (4·8) | 12 (5·7) | 14 (1·4) | 12 (1·2) | 24 (2·0) | 24 (2·0) |
| | Total | 210 | 210 | 966 | 966 | 1176 | 1176 |

TABLE II.—*Risks of breast cancer in women using oral contraceptives before first term pregnancy, relative to those in women not doing so**

| Parity | Total duration OC use (months) | Age up to 35 yrs | Age 36+ | All ages |
|---|---|---|---|---|
| 0 | 0 | — | 1·00 | 1·00 |
| | 1–12 | — | 0·48 | 0·50 |
| | 13–48 | — | 1·39 | 1·00 |
| | 49 or more | — | 0·74 | 0·69 |
| 1 or more | 0 | 1·00 | 1·00 | 1·00 |
| | 1–12 | 0·77 | 0·59 | 0·79 |
| | 13–48 | 0·59 | 1·38 | 0·70 |
| | 49 or more | 0·67 | 1·27 | 0·90 |
| All parities | 0 | 1·00 | 1·00 | 1·00 |
| | 1–12 | 0·74 | 0·59 | 0·70 (0·43–1·12)† |
| | 13–48 | 0·56 | 1·23 | 0·81 (0·47–1·39) |
| | 49 or more | 0·55 | 0·94 | 0·81 (0·43–1·53) |

* Adjusted for effects of social class, age at menarché, age at first term pregnancy, menopausal state, smoking habits, history of breast biopsy and family history of breast cancer.

† 95% confidence limits.

TABLE III.—*Miscarriage or termination of pregnancy before first term pregnancy in women with breast cancer and matched controls (percentages in parentheses)*

| Parity | Miscarriage/ termination | Age up to 35 yrs: Breast Ca. | Age up to 35 yrs: Control | Age 36 yrs+: Breast Ca. | Age 36 yrs+: Control | All ages: Breast Ca. | All ages: Control |
|---|---|---|---|---|---|---|---|
| 0 | No | 17 (81·0) | 19 (90·5) | 95 (89·6) | 88 (83·0) | 112 (88·2) | 107 (84·3) |
| | Yes | 4 (19·0) | 2 (9·5) | 11 (10·4) | 18 (17·0) | 15 (11·8) | 20 (15·7) |
| | Total | 21 | 21 | 106 | 106 | 127 | 127 |
| 1 or more | No | 176 (93·1) | 170 (89·9) | 775 (90·1) | 772 (89·8) | 951 (90·7) | 942 (89·8) |
| | Yes | 13 (6·9) | 19 (10·1) | 85 (9·9) | 88 (10·2) | 98 (9·3) | 107 (10·2) |
| | Total | 189 | 189 | 860 | 860 | 1049 | 1049 |
| All parities | No | 193 (91·9) | 189 (90·0) | 870 (90·1) | 860 (89·0) | 1063 (90·4) | 1049 (89·2) |
| | Yes | 17 (8·1) | 21 (10·0) | 96 (9·9) | 106 (11·0) | 113 (9·6) | 127 (10·8) |
| | Total | 210 | 210 | 966 | 966 | 1176 | 1176 |

factory, as the probability of obtaining such diverse results by chance alone is small. Other explanations can be derived from differences in the material studied and, perhaps, in the methods of analysis. These differences include at least 5 that are potentially important.

First, Pike *et al.* (1981) initially identified 245 living patients as being eligible for their study, but eventually succeeded in including only 163 (67%) in the analysis. Difficulties were also experienced in assembling the 2 control series (made up of "neighbourhood" controls and "school-friend" controls). It seems possible that this incomplete response might have affected their findings. Secondly, relatively few of the British women had used OC at all before their first term pregnancy (23% of the control women aged up to 35 years in the period 1968–80, as against 48% in the Californian series in the period 1972–78) and very few had used them for more than 4 years (5·7% against 9·6%). If the risk of breast cancer increases sharply with duration of use, the number of British women at substantial risk must have been very small and even proportionately small within the group that had used OC for 4 years or more. Thirdly, our series of young women included subjects up to 35 years of age (because of our matching criteria), whereas the cut-off age in the Californian series was 32. It is conceivable (though unlikely) that OC use before first term pregnancy affects the incidence of breast cancer only in very young women, and that its effect was diluted in our series by extending the age limit to 35. Fourthly, whereas the Californian series included women who were interviewed several years after their cancer was diagnosed, our series did not. Previous studies have found that the survival rate tends to be higher in women taking OC at the time of diagnosis than in others (Spencer *et al.*, 1978; Vessey *et al.*, 1979) and, if this is generally true, it would tend to produce a spurious association between OC use and breast cancer in any series that favoured long-term survivors. Pike *et al.* (1981) "investigated this possible bias by dividing cases into strata depending on the length of time from diagnosis to interview, but found no evidence that this influenced the OC finding". This, however, does not wholly eliminate the possibility, as the subjects were diagnosed over a 6-year period (1972–78) during which the use of OC in the population might be expected to have increased. Finally, the RRs in the 2 series were standardized in different ways. Pike *et al.* (1981) examined the effects of age at menarché, family history of breast cancer and whether or not women had had a full-term pregnancy, and found that the RR associated with OC use was "hardly altered" by adjusting for them (whether singly or in combination is unclear). They did not, however, take account of age at first term pregnancy, as there was no clear trend in the risk with this variable. The RR of breast cancer was, however, materially less when first term pregnancy occurred under 25 years of age (under 20 years 0·81, 20–24 years 0·67) and this must have increased the period at risk of OC use in the breast-cancer patients compared with the controls. In our series, in contrast, the RRs were standardized (though crudely) for 7 factors relating to the risk of breast cancer and OC use (age at menarché, age at first term pregnancy, history of breast biopsy, family history of breast cancer, social class, smoking habits, and—among women aged 36 or more—menopausal status). This standardization materially reduced the estimates of RR associated with long-term use of the drugs. It must be stressed, however, that even without such standardization, the RR for OC use for 4 years or more remains $<1$ for the young group.

Summing up, we suspect that the disparities between the results obtained in Britain and California are due to the combined effect of chance, selective factors associated with the availability of subjects for interview, and (perhaps)

TABLE IV.—*Risks of breast cancer in women experiencing a miscarriage or termination of pregnancy before first term pregnancy relative to those in women not doing so**

| Parity | Miscarriage/ termination | Age up to 35 yrs | Age 36 yrs or more | All ages |
|---|---|---|---|---|
| 0 | No | — | 1·00 | 1·00 |
| | Yes | — | 0·62 | 0·73 |
| 1 or more | No | 1·00 | 1·00 | 1·00 |
| | Yes | 0·62 | 0·84 | 0·84 |
| All parities | No | 1·00 | 1·00 | 1·00 |
| | Yes | 0·79 | 0·82 | 0·84 (0·63–1·12)† |

* Adjusted for effects of social class, age at menarché, age at first term pregnancy, menopausal state, smoking habits, history of breast biopsy and family history of breast cancer.
† 95% Confidence limits.

differences in the method of analysis. We are encouraged to think that, when more data are available and the effect of random variation is reduced, the results will not show any effect of OC use before first term pregnancy on the incidence of breast cancer in young women by: (i) the consistency of the negative findings in our series at ages up to 35 years and at 36 to 50 years and (ii) the lack of any suggestion of an effect in the few cases so far observed in the cohort being studied in Oxford in conjunction with the Family Planning Association (Vessey *et al.*, 1981). We note, however, that the Californian series related to a population in which OC had been used for long periods by more women than in Britain, and for this reason our negative results carry less weight than would appear at first glance. More data will, therefore, be needed relating to the period before first term pregnancy for both OC use and first-trimester abortion (on which the 2 series also differed) before any firm conclusion can be reached.

We thank the medical staff at the participating hospitals for allowing us to study patients under their care, and Miss Keena Jones, Mrs Moya Simmonds, Mrs Judith Young, Mrs Kate Rodriguez, and Miss Ruth Harris for help in conducting the interviews and following up the patients. We are also grateful to the Medical Research Council and the Imperial Cancer Research Fund for financial support.

### REFERENCES

BOSTON COLLABORATIVE DRUG SURVEILLANCE PROGRAM (1973) Oral contraceptives and venous thromboembolic disease, surgically confirmed gall-bladder disease and breast tumours. *Lancet*, i, 1399.

BRESLOW, N. E., DAY, N. E., HALVORSEN, K. T., PRENTICE, R. L. & SABAI, C. (1978) Estimation of multiple relative risk functions in matched case-control studies. *Am. J. Epidemiol.*, **108**, 299.

PIKE, M. C., HENDERSON, B. E., CASAGRANDE, J. T., ROSARIO, I. & GRAY, G. E. (1981) Oral contraceptive use and early abortion as risk factors for breast cancer in young women. *Br. J. Cancer*, **43**, 72.

SPENCER, J. D., MILLIS, R. R. & HAYWARD, J. L. (1978) Contraceptive steroids and breast cancer. *Br. Med. J.*, i, 1024.

VESSEY, M. P., DOLL, R. & SUTTON, P. M. (1972) Oral contraceptives and breast neoplasia: A retrospective study. *Br. Med. J.*, iii, 719.

VESSEY, M. P., DOLL, R. & JONES, K. (1975) Oral contraceptives and breast cancer: Progress report of an epidemiological study. *Lancet*, i, 941.

VESSEY, M. P., DOLL, R., JONES, K., MCPHERSON, K. & YEATES, D. (1979) An epidemiological study of oral contraceptives and breast cancer. *Br. Med. J.*, i, 1755.

VESSEY, M. P., MCPHERSON, K. & DOLL, R. (1981) Breast cancer and oral contraceptives: Findings in the Oxford-Family Planning Association contraceptive study. *Br. Med. J.*, **282**, 2093.